# Supplementary material for: Identifying novel genetic variants in epidermolysis Bullosa among Middle Eastern Arab Families: Insights from whole exome sequencing and computational analysis
Source: PLoS One. 2025 Sep 16;20(9):e0328296. doi: 10.1371/journal.pone.0328296 (PMC12440221; doi:10.1371/journal.pone.0328296)
Supplement: S1 Table — (DOCX) [file pone.0328296.s001.docx]

**S1 Table**: Primer details used in PCR and Sanger sequencing**.**

| Family ID | Gene_ Mutation | Forward Primer (5`- 3`) | Reverse Primer (5`- 3`) |
| --- | --- | --- | --- |
| 1 | COL7A1_c.5924_5927del | GAGAGAATGCTGGTGGCTGT | TAGTGGTGCCCACAGGCATA |
| 2 | COL7A1_c.5924_5927del | GAGAGAATGCTGGTGGCTGT | TAGTGGTGCCCACAGGCATA |
| 3 | COL7A1_c.5888 G>A | GCCACTTCTGCTCACCTCCT | TCAAGGTGGGTTGTTTAGGG |
| 4 | COL7A1_c.1633C>T | GGATAACGAGACAGGGAGGA | CTGCCTGTGAGCCCTGTAA |
| 5 | COL7A1_c.4448G>A | CGCCCTGATGGAAAAGAAG | GTCAGAGGTCGTGGTTTTGG |
| 6 | COL17A1_c.1394G>A | GGTAACAAGGGTCTGCACCA | AGAGAATCTGGAGGGAAAAGG |
| 7 | LAMB3_c.1977-1G>A | TTCAAACTGCTCCCTCTTCC | GGGAGGAGAATTGGGAATGT |
| 8 | COL7A1 _c.8305-1G>A | CAGGGACTATGGTGAGACTGC | TCACTTGGTCCCTGTGTCTG |
| 9 | COL7A1_c.6268_6269del | ACAAGGTCACAGGGGAGAGA | ACCCGCTATTTGCATTTCAG |
| 10 | COL7A1_c.2005C/T | AGGGAGTGGGATTCTGAAGG | ATTTCGGATTAGCTGGAGCA |
|  | COL7A1_c.8245G/A | GCCCCTATGTGCAACAGAT | CCACCTGGCAGTGTTGGT |
| 11 | COL7A1 c.1837C>T | TAGAGTCTGGGGGCAGTGTC | TGAGTACTGCAGGAGGCTTG |
| 12 | COL7A1 c.6751-1G>A | GTGACCCCTATGGCAGAGC | GGCCCATGTTCTCTCATGTC |
