# Supplementary material for: Identifying novel genetic variants in epidermolysis Bullosa among Middle Eastern Arab Families: Insights from whole exome sequencing and computational analysis
Source: PLoS One. 2025 Sep 16;20(9):e0328296. doi: 10.1371/journal.pone.0328296 (PMC12440221; doi:10.1371/journal.pone.0328296)
Supplement: S2 Table — (DOCX) [file pone.0328296.s002.docx]

**S2 Table:** Clinical complications across multiple systems in index cases from Saudi EB families.

| Family ID | **Pedigree ID** | Genitourinary | Upper Airway | Pseudosyndactly | Gastrointestinal | Ophthalmological | Cardiology | Anemia (iron deficiency) | Skin Cancer | Infections |
| --- | --- | --- | --- | --- | --- | --- | --- | --- | --- | --- |
| 1 | III-2 | No | No | No | Dysphagia | Photophobia | No | No | No | Wound Infection |
| 1 | III-1 | No | No | Yes (toes) | Dysphagia | photophobia | No | Yes | No | Wound Infection |
| 2 | IV-6 | No | No | Yes (toes) | Dysphagia | Dryness | No | Yes | No | Wound Infection |
| 2 | III-15 | No | No | Yes (both) | No | Dryness | No | Yes | Yes | Wound Infection |
| 2 | III-16 | No | No | Yes (both) | No | Dryness | No | Yes | No | Wound Infection |
| 2 | III-19 | No | No | Yes (both) | Dysphagia | Dryness | No | Yes | No | Wound Infection |
| 3 | II-5 | No | No | Yes (both) | Dysphagia | Photophobia | No | Yes | No | Wound Infection |
| 4 | III-2 | No | No | Yes (both) | Dysphagia, esophageal strictures | Photophobia | No | Yes | No | Wound Infection |
| 5 | II-7 | No | No | No | Dysphagia |  | No |  | No | Wound Infection |
| 6 | II-1 | No | No | No | Dysphagia, esophageal stricture | Photophobia | No | No | No | Wound Infection |
| 7 | II-1 | Yes, Bliste, diaper rash esp in testicles | No | No | No | corneal abrasion, photophobia | No | No | No | Wound Infection |
| 8 | II-2 | No | No | Yes (toes) | Dysphagia | photophobia, Dryness | No | ? | No | Wound Infection |
| 9 | II-1 | Yes, urethral obstruction | No | No | No | corneal abrasion | No | Yes | No | Wound Infection |
| 10 | II-4 | Yes, Repeated diaper rash | No | Yes (both) | No | Photophobia | No | Yes | No | Wound Infection |
| 11 | III-7​ | No | No | Yes (both) | Dysphagia | photophobia | No | No | No | Wound Infection |
| 12 | II-3 | No | No | Yes (toes) | dysphagia, esophageal stricture | Dryness | No | Yes | No | Wound Infection |
