# Supplementary material for: Identifying novel genetic variants in epidermolysis Bullosa among Middle Eastern Arab Families: Insights from whole exome sequencing and computational analysis
Source: PLoS One. 2025 Sep 16;20(9):e0328296. doi: 10.1371/journal.pone.0328296 (PMC12440221; doi:10.1371/journal.pone.0328296)
Supplement: S3 Table — (DOCX) [file pone.0328296.s003.docx]

**S3 Table:** Results of the segregation analysis for the identified variants in affected and unaffected family members.

| **Family ID** | **Gene: variant** | **Pedigree ID** | | **Zygosity** | **Genotyping** | **Variant Mode of Inheritance** |
| --- | --- | --- | --- | --- | --- | --- |
| 1 | *COL7A1* (NM_000094.4), c.5924_5927del (p. Glu1975Glyfs*29) | Index | III-1 | **Homozygous Mutant** | **_/_** | Autosomal Recessive |
|  |  | Aunt | II-4 | Heterozygous | AACG/_ |  |
|  |  | Uncle | II-5 | Homozygous Normal | AACG/AACG |  |
|  |  | Brother | III-2 | Homozygous Mutant | _/_ |  |
| 2 | *COL7A1* (NM_000094.4), c.5924_5927del  (p. Glu1975Glyfs*29) | Index | IV-6 | **Homozygous Mutant** | _/_ | Autosomal Recessive |
|  |  | Father | III-11 | Heterozygous | AACG/_ |  |
|  |  | Mother | III-5 | Heterozygous | AACG/_ |  |
|  |  | Aunt | III-7 | Homozygous Normal | AACG/AACG |  |
|  |  | Uncle | III-15 | Homozygous Mutant | _/_ |  |
|  |  | Uncle | III-16 | Homozygous Mutant | _/_ |  |
|  |  | Uncle | III-19 | Homozygous Mutant | _/_ |  |
|  |  | Aunt | III-8 | Homozygous Normal | AACG/AACG |  |
|  |  | Uncle | III-12 | Heterozygous | AACG/_ |  |
|  |  | Aunt | III-13 | Homozygous Normal | AACG/AACG |  |
|  |  | Uncle | III-9 | Heterozygous | AACG/_ |  |
|  |  | Brother | IV-4 | Heterozygous | AACG/_ |  |
| 3 | *COL7A1* (NM_000094.4), c.5888 G>A  (p. Trp19638*) | Index | II-5 | **Homozygous Mutant** | **A/A** | Autosomal Recessive |
|  |  | Sister | II-1 | Homozygous Normal | G/G |  |
|  |  | Sister | II-3 | Heterozygous | G/A |  |
|  |  | Sister | II-6 | Homozygous Normal | G/G |  |
|  |  | Sister | II-7 | Homozygous Normal | G/G |  |
| 4 | *COL7A1*(NM_000094.4), c.1633C>T (p. Gln545*) | Index | III-2 | **Homozygous Mutant** | T/T | Autosomal Recessive |
|  |  | Mother | II-1 | Heterozygous | C/T |  |
|  |  | Father | II-2 | Heterozygous | C/T |  |
|  |  | Sister | III-1 | Heterozygous | C/T |  |
| 5 | *COL7A1* (NM_000094.4), c.4448G>A  (p. Gly1483Asp) | Index | II-7 | **Homozygous Mutant** | A/A | Autosomal Recessive |
|  |  | Brother | II-4 | Heterozygous | G/A |  |
|  |  | Brother | II-5 | Homozygous Normal | G/G |  |
|  |  | Brother | II-6 | Homozygous Normal | G/G |  |
| 6 | *COL17A1* (NM_000494.4), c.1394G>A  (p. Trp465*) | Index | II-1 | **Homozygous Mutant** | A/A | Autosomal Recessive |
|  |  | Sister | II-2 | Homozygous Normal | G/G |  |
|  |  | Sister | II-3 | Heterozygous | G/A |  |
|  |  | Brother | II-4 | Heterozygous | G/A |  |
|  |  | Brother | II-5 | Homozygous Normal | G/G |  |
|  |  | Sister | II-6 | Homozygous Normal | G/G |  |
| 7 | *LAMB3* (NM_000228.3), c.1977-1G>A | Index | II-1 | **Homozygous Mutant** | A/A | Autosomal Recessive |
|  |  | Mother | I-1 | Heterozygous | G/A |  |
|  |  | Father | I-2 | Heterozygous | G/A |  |
|  |  | Brother | II-2 | Heterozygous | G/A |  |
| 8 | *COL7A1* (NM_000094.4), c.8305-1G>A | Index | II-2 | **Homozygous Mutant** | A/A | Autosomal Recessive |
|  |  | Sister | II-1 | Heterozygous | G/A |  |
| 9 | *COL7A1* (NM_000094.4), c.6268_6269del  (p. Pro2090Trpfs*8) | Mother | I-1 | Heterozygous | GG/_ | Autosomal Recessive |
|  |  | Index | II-1 | **Homozygous Mutant** | _/_ |  |
|  |  | Father | I-2 | Heterozygous | GG/_ |  |
| 10 | *COL7A1*(NM_000094.4),  c.2005C>T(p.Argg669*) and  *COL7A1*(NM_000094.4), c.8245G>A  (p. Gly2749Arg) | Index | II-12 | **Compound Heterozygous** | C/T and G/A | Compound Heterozygosity |
|  |  | Father | I-2 | Heterozygous | C/T |  |
|  |  | Mother | I-3 | Heterozygous | G/A |  |
|  |  | Half-Brother | II-5 | Heterozygous | C/T |  |
|  |  | Half-Brother | II-9 | Homozygous Normal | C/C |  |
|  |  | Half-Sister | II-7 | Homozygous Normal | C/C |  |
| 11 | *COL7A1* (NM_000094.4), c.1837C>T  (p. Arg613*) | Index | III-7​ | **Homozygous Mutant** | T/T | Autosomal Recessive |
|  |  | Sister | III-2 | Heterozygous | C/T |  |
|  |  | Sister | III-3​ | Heterozygous | C/T |  |
|  |  | Sister | III-4 | Heterozygous | C/T |  |
|  |  | Uncle | II-7​ | Heterozygous | C/T |  |
|  |  | Aunt | II-5​ | Heterozygous | C/T |  |
|  |  | Cousin | III-14​ | **Homozygous Mutant** | T/T |  |
| 12 | *COL7A1*: c.6751-1G>A | Index | II-3 | **Heterozygous Mutant** | G/A | Autosomal Dominant |
|  |  | Brother | II-1 | Homozygous Normal | G/G |  |
