# Supplementary material for: Identifying novel genetic variants in epidermolysis Bullosa among Middle Eastern Arab Families: Insights from whole exome sequencing and computational analysis
Source: PLoS One. 2025 Sep 16;20(9):e0328296. doi: 10.1371/journal.pone.0328296 (PMC12440221; doi:10.1371/journal.pone.0328296)
Supplement: Attachment — Submitted filename: EB rebuttal 120525.docx [file pone.0328296.s005.docx]

**To the Editor and Reviewers,**

**We would like to extend our sincere thanks to the Editor and all three reviewers for their thoughtful and constructive feedback on our manuscript entitled:
"Identifying Novel Genetic Variants in Epidermolysis Bullosa Among Middle Eastern Arab Families: Insights from Whole Exome Sequencing and Computational Analysis."**

**We greatly appreciate the time and effort you have invested in reviewing our work. Your detailed comments have helped us significantly improve the quality, clarity, and scientific rigor of the manuscript. We have carefully addressed each point raised and revised the manuscript accordingly. All changes made to the manuscript have been highlighted, and we have provided a detailed point-by-point response below.**

**We are grateful for the opportunity to revise and resubmit our work, and we hope that the revised version meets your expectations.**

**Editor Comments:**

**The findings of the manuscript provide valuable insights on   molecular basis of Epidermolysis Bullosa (EB) from Middle eastern families. The study  reports novel and known pathogenic mutations in three genes (*COL7A1*, *COL17A1*, *LAMB3*)  from  12 families affected with EB by employing exome sequencing. The manuscript is well structured technically sound. However the manuscript requires minor reversions for improvements of quality and better comprehension before acceptance for publication. The following points needs to be considered:**

1. **As the study reports families from Saudi Arabia, Yemen and Syria, so the title referring ‘ Saudi Arabian families’ is misleading. The use of  term ‘Middle eastern’ would have been more appropriate.**

**Response:** Thank you for your valuable comment. Based on the reviewer's comment, we amended the title and text to include Middle Eastern Arab families.

2. **The quality of pedigrees in  figure 1 needs considerable  improvements for clarity.
   Response:** Thank you for your valuable comment. Figure 1 has been revised to enhance clarity and readability, ensuring that the structure and genotype information are presented more clearly to the readers.
3. **A  flow chart showing  exome sequencing pipeline will be useful.**

**Response:** Thank you for the suggestion. We have now included a flowchart showing the exome sequencing pipeline in the revised manuscript (see Figure 1).

4. **More Information on inclusion and diagnostic criteria, details on clinical phenotypes should be added.**

**Response:** Thank you for your comment. We have now included detailed information on the inclusion and diagnostic criteria and clinical phenotype descriptions in the Methods section of the revised manuscript.

5. **Some gene mutation nomenclature should be checked, especially the variant described at the protein level. Please follow the guidelines set by the Human Genome Variation Society (HGVS).**

**Response:** Thank you for your valuable observation regarding the gene mutation nomenclature. In response, we have carefully reviewed all listed variants, particularly those described at the protein level, to ensure full compliance with the Human Genome Variation Society (HGVS) guidelines.

The following variants have been checked and formatted according to HGVS recommendations:

- COL7A1, c.5924_5927del (p.Glu1975Glyfs*29)
- COL7A1, c.5888G>A (p.Trp1963*)
- COL7A1, c.1633C>T (p.Gln545*)
- COL17A1, c.1394G>A (p.Trp465*)
- COL7A1, c.6268_6269del (p.Pro2090Trpfs*8)
- COL7A1, c.2005C>T (p.Arg669*)
- COL7A1, c.1837C>T (p.Arg613*)

All mutations now follow the correct HGVS format:

- Protein-level descriptions begin with "p." (without a space),
- Amino acid changes use the three-letter code,
- Frameshift mutations specify the new amino acid (if known), followed by "fs" and the predicted stop codon position (e.g., fs*8),
- Stop codons are represented by an asterisk * as per HGVS standards.

We appreciate your input and have ensured the nomenclature is accurate and standardized throughout.

6. **Functional consequences of splice site and missense variant should be discussed in discussion.**

**Response:** Thank you for the comment. We have now included a brief discussion of the functional consequences of the splice site and missense variants in the revised Discussion section.

7. **There are grammatical and typological errors in different sections of the manuscript which needs to be corrected**

**Response:** Thank you for your valuable comment. All grammatical and typographical errors have been corrected throughout the manuscript.

**Journal Requirements:**

1. **When submitting your revision, we need you to address these additional requirements.**

**Please ensure that your manuscript meets PLOS ONE's style requirements, including those for file naming. The PLOS ONE style templates can be found at**

[**https://journals.plos.org/plosone/s/file?id=wjVg/PLOSOne_formatting_sample_main_body. pdf**](https://journals.plos.org/plosone/s/file?id=wjVg/PLOSOne_formatting_sample_main_body.%20pdf) **and** [**https://journals.plos.org/plosone/s/file?id=ba62/PLOSOne_formatting_sample_title_authors_affiliations.pdf**](https://journals.plos.org/plosone/s/file?id=ba62/PLOSOne_formatting_sample_title_authors_affiliations.pdf)

**Response:** Thank you for your comment. We have revised the manuscript formatting and file naming to fully comply with PLOS ONE’s style requirements.

2. **We suggest you thoroughly copyedit your manuscript for language usage, spelling, and grammar. If you do not know anyone who can help you do this, you may wish to consider employing a professional scientific editing service.**

**The American Journal Experts (AJE) (**[**https://www.aje.com/**](https://urldefense.com/v3/__https:/www.aje.com/__;!!CvxqaX2N8Ww!xxefLDJac4RLWSYbFX1jSWhGnFy9Mb3a2WLA4qd_dQiUERat-qKiLdeyNNnUD_afpWbrmGBanzUy6Bd04q5ygcA$)**) is one such service that has extensive experience helping authors meet PLOS guidelines and can provide language editing, translation, manuscript formatting, and figure formatting to ensure your manuscript meets our submission guidelines. Please note that having the manuscript copyedited by AJE or any other editing services does not guarantee selection for peer review or acceptance for publication.**

**Upon resubmission, please provide the following:**

**-The name of the colleague or the details of the professional service that edited your manuscript**

**-A copy of your manuscript showing your changes by either highlighting them or using track changes (uploaded as a *supporting information* file)**

**-A clean copy of the edited manuscript (uploaded as the new *manuscript* file)**

**Response:** Thank you for your valuable comment. We have thoroughly revised the manuscript for language, spelling, and grammar. It was also proofread by English language professors from the Department of English, Faculty of Arts and Humanities, King Abdulaziz University, Saudi Arabia.

3. **Please provide additional details regarding participant consent. In the ethics statement in the Methods and online submission information, please ensure that you have specified what type you obtained (for instance, written or verbal, and if verbal, how it was documented and witnessed). If your study included minors, state whether you obtained consent from parents or guardians. If the need for consent was waived by the ethics committee, please include this information.**

**Once you have amended this/these statement(s) in the Methods section of the manuscript, please add the same text to the “Ethics Statement” field of the submission form (via “Edit Submission”). For additional information about PLOS ONE ethical requirements for human subjects research, please refer to** [**http://journals.plos.org/plosone/s/submission-guidelines#loc-human-subjects-research**](https://urldefense.com/v3/__http:/journals.plos.org/plosone/s/submission-guidelines*loc-human-subjects-research__;Iw!!CvxqaX2N8Ww!xxefLDJac4RLWSYbFX1jSWhGnFy9Mb3a2WLA4qd_dQiUERat-qKiLdeyNNnUD_afpWbrmGBanzUy6Bd0zPp5aoE$)**.**

**Response:** Thank you for your valuable comment. Written informed consent was obtained from all participants, and this has been stated in the Methods section.

4. **Thank you for stating in your Funding Statement:**

**Please provide an amended statement that declares *all* the funding or sources of support (whether external or internal to your organization) received during this study, as detailed online in our guide for authors at** [**http://journals.plos.org/plosone/s/submit-now**](https://urldefense.com/v3/__http:/journals.plos.org/plosone/s/submit-now__;!!CvxqaX2N8Ww!xxefLDJac4RLWSYbFX1jSWhGnFy9Mb3a2WLA4qd_dQiUERat-qKiLdeyNNnUD_afpWbrmGBanzUy6Bd0VbindQs$)**.  Please also include the statement “There was no additional external funding received for this study.” in your updated Funding Statement.**

**Please include your amended Funding Statement within your cover letter. We will change the online submission form on your behalf.**

**Response:** Thank you for your valuable comment. The authors would also like to thank the Saudi Society for Laboratory Medicine for their scientific support.

5. **Thank you for stating the following in the Acknowledgments Section of your manuscript:**

**We note that you have provided funding information that is not currently declared in your Funding Statement. However, funding information should not appear in the Acknowledgments section or other areas of your manuscript. We will only publish funding information present in the Funding Statement section of the online submission form.**

- **Please remove any funding-related text from the manuscript and let us know how you would like to update your Funding Statement. Currently, your Funding Statement reads as follows:**
- **This publication is based on work supported by The Saudi Society for Laboratory Medicine for funding the research.**
- **Please include your amended statements within your cover letter; we will change the online submission form on your behalf.**

**Response:** Thank you for your valuable comment. The funding statement has been removed from the Acknowledgments section as follows:
“The authors would also like to thank the Saudi Society for Laboratory Medicine for their scientific support. There was no additional external funding received for this study.

6. **We note that there is identifying data in the Supporting Information file “EB Supplementary Tables”. Due to the inclusion of these potentially identifying data, we have removed this file from your file inventory. Prior to sharing human research participant data, authors should consult with an ethics committee to ensure data are shared in accordance with participant consent and all applicable local laws.**

**Data sharing should never compromise participant privacy. It is therefore not appropriate to publicly share personally identifiable data on human research participants. The following are examples of data that should not be shared:**

**-Name, initials, physical address**

**-Ages more specific than whole numbers**

**-Internet protocol (IP) address**

**-Specific dates (birth dates, death dates, examination dates, etc.)**

**-Contact information such as phone number or email address**

**-Location data**

**-ID numbers that seem specific (long numbers, include initials, titled “Hospital ID”) rather than random (small numbers in numerical order)**

**Data that are not directly identifying may also be inappropriate to share, as in combination they can become identifying. For example, data collected from a small group of participants, vulnerable populations, or private groups should not be shared if they involve indirect identifiers (such as sex, ethnicity, location, etc.) that may risk the identification of study participants.**

**Additional guidance on preparing raw data for publication can be found in our Data Policy (**[**https://journals.plos.org/plosone/s/data-availability#loc-human-research-participant-data-and-other-sensitive-data**](https://urldefense.com/v3/__https:/journals.plos.org/plosone/s/data-availability*loc-human-research-participant-data-and-other-sensitive-data__;Iw!!CvxqaX2N8Ww!xxefLDJac4RLWSYbFX1jSWhGnFy9Mb3a2WLA4qd_dQiUERat-qKiLdeyNNnUD_afpWbrmGBanzUy6Bd0Y21VBKA$)**) and in the following article:** [**http://www.bmj.com/content/340/bmj.c181.long**](https://urldefense.com/v3/__http:/www.bmj.com/content/340/bmj.c181.long__;!!CvxqaX2N8Ww!xxefLDJac4RLWSYbFX1jSWhGnFy9Mb3a2WLA4qd_dQiUERat-qKiLdeyNNnUD_afpWbrmGBanzUy6Bd0IJE3n80$)**.**

**Please remove or anonymize all personal information (<specific identifying information in file to be removed>), ensure that the data shared are in accordance with participant consent, and re-upload a fully anonymized data set. Please note that spreadsheet columns with personal information must be removed and not hidden as all hidden columns will appear in the published file.**

**Response:**
Thank you for your guidance. As this is a genetic study, only non-identifiable information such as age (in whole years) and gender was included, as these variables are essential for assessing disease presentation and risk profiles. No personally identifiable information (e.g., names, addresses, ID numbers) was shared. The data were approved for use and sharing in this format by our institutional ethics committee.

7. **PLOS requires an ORCID iD for the corresponding author in Editorial Manager on papers submitted after December 6th, 2016. Please ensure that you have an ORCID iD and that it is validated in Editorial Manager. To do this, go to ‘Update my Information’ (in the upper left-hand corner of the main menu), and click on the Fetch/Validate link next to the ORCID field. This will take you to the ORCID site and allow you to create a new iD or authenticate a pre-existing iD in Editorial Manager.**

**Response:** Thank you for your valuable comment. Yes, the corresponding author and co-authors have validated ORCID iDs as listed below:

- Nancy Shehata: <https://orcid.org/0009-0006-0237-3478>
- Noor Ahmad: <https://orcid.org/0000-0002-7133-656X>
- Hadiah Bassam Al Mahdi: <https://orcid.org/0000-0003-3483-9499>
- Shmoukh Abdullah Alghuraibi: <https://orcid.org/0009-0005-5552-851X>

8. **Your ethics statement should only appear in the Methods section of your manuscript. If your ethics statement is written in any section besides the Methods, please delete it from any other section.**

**Response:** We appreciate the reviewer’s observation. In compliance with the journal’s guidelines, the ethics statement has been retained exclusively in the Methods section and removed from all other parts of the manuscript.

9. **Please upload a new copy of Figure 1 as the detail is not clear. Please follow the link for more information:** [**https://blogs.plos.org/plos/2019/06/looking-good-tips-for-creating-your-plos-figures-graphics/**](https://urldefense.com/v3/__https:/blogs.plos.org/plos/2019/06/looking-good-tips-for-creating-your-plos-figures-graphics/__;!!CvxqaX2N8Ww!xxefLDJac4RLWSYbFX1jSWhGnFy9Mb3a2WLA4qd_dQiUERat-qKiLdeyNNnUD_afpWbrmGBanzUy6Bd093Vnu8s$)**"** [**https://blogs.plos.org/plos/2019/06/looking-good-tips-for-creating-your-plos-figures-graphics/**](https://urldefense.com/v3/__https:/blogs.plos.org/plos/2019/06/looking-good-tips-for-creating-your-plos-figures-graphics/__;!!CvxqaX2N8Ww!xxefLDJac4RLWSYbFX1jSWhGnFy9Mb3a2WLA4qd_dQiUERat-qKiLdeyNNnUD_afpWbrmGBanzUy6Bd093Vnu8s$)

**Response:** Thank you for your valuable comment. Figure 1 has been revised to enhance clarity and readability, ensuring that the structure and genotype information are presented more clearly to the readers. Please note that with the addition of the figure illustrating the WES workflow, the order of the figures has been adjusted accordingly.

10. **Please review your reference list to ensure that it is complete and correct. If you have cited papers that have been retracted, please include the rationale for doing so in the manuscript text, or remove these references and replace them with relevant current references. Any changes to the reference list should be mentioned in the rebuttal letter that accompanies your revised manuscript. If you need to cite a retracted article, indicate the article’s retracted status in the References list and also include a citation and full reference for the retraction notice.**

**Response:** Thank you for your comment. As requested, we have reviewed and updated the reference list to ensure it is complete and accurate. All newly added references have been included in the revised manuscript and this rebuttal letter. We also confirm that no retracted articles are cited.

**Reviewers:**

**Reviewer #1: This manuscript will make a nice contribution to the field of human genetics. But needs some improvements and minor revisions before formal acceptance for publication in PlosOne. Authors should consider following points during minor revisions:**
**1. Authors must provide the transcript IDs of different genes in which mutations have been identified.**

**Response:** Thank you for your insightful comment. We have now included the transcript IDs (RefSeq accession numbers) for all genes in which mutations were identified, ensuring that each variant is accurately referenced according to the correct transcript. This information has been added to the relevant sections of the manuscript and tables, as applicable.

**2****. In the abstract there are empty parentheses "()".**

**Response:** Thank you for your valuable comment. The empty parentheses in the abstract have been addressed by adding the appropriate variant information to ensure completeness and clarity.

**3. Quality of all the figures needs improvement.**

**Response:** Thank you for the valuable suggestion. We have revised and enhanced the quality of all figures to improve clarity and readability. Additionally, we have included a new flowchart illustrating the exome sequencing pipeline in the revised manuscript (see **Figure 1**).
**4. A figure detailing the flow chart of exome sequencing should be made for clear understanding.**

**Response**: Thank you for the suggestion. We have now included a flowchart showing the exome sequencing pipeline in the revised manuscript (see **Figure 1**).
5. **There are some splice variants, these splice variants should be analyzed using bioinformatics softwares like NetGene2 etc. for assessing the effect of these variants. And graphical representation in the form of a a figure showing intron inclusion in the final protein or exon skipping due to aberrant splicing should be given.**

**Response:** We appreciate the reviewer’s suggestion regarding the analysis of splice variants using tools such as NetGene2. In our study, splice variant prediction was already performed using **SpliceAI**, a deep learning-based tool developed by Illumina, which has demonstrated superior accuracy in predicting splicing alterations compared to traditional algorithms like NetGene2.

SpliceAI offers several advantages:

- It considers long-range dependencies in genomic sequences (up to 10,000 bp context),
- Provides delta scores reflecting the probability of gain or loss of splice sites,
- Has been extensively benchmarked and validated against experimentally confirmed splice-altering variants.

Given its robust performance and reliability, we chose SpliceAI to ensure comprehensive and accurate splice impact prediction. However, we are open to including results from additional tools such as NetGene2 for comparative purposes if required.

We have included the figures of splice site infographics in Figure 5.

6. **Carefully check the grammar and other typographical mistakes before resubmission.**

**Response:** Thank you for the note. We have carefully reviewed and corrected all grammatical and typographical errors throughout the manuscript before resubmission.

**Reviewer #2: The study presents significant findings on novel and recurrent mutations in EB among Saudi families, contributing valuable data on a region with limited molecular epidemiological studies. The manuscript is well-structured and follows a logical flow, but some sections require refinement for clarity and conciseness.**

**Specific Comments:
Title & Abstract**

**- The title is appropriate but could be more specific by highlighting "molecular findings" or "genetic profiling."**

**Response:** Thank you for your valuable comment. We are unable to add variant descriptions in the title as they many variants.

- **The abstract summarizes key findings well, but some sentences are wordy. Consider shortening complex phrases for better readability.**

- **The Results section should clearly differentiate between novel and recurrent mutations in the abstract.**

**- The conclusion should explicitly state the clinical significance of the study.**

**Response:** Thank you for your valuable comment. We revised the title, abstract, results, and conclusion of the study in the revised manuscript.

**Introduction
- The introduction provides good background information, but the prevalence statistics on EB in Saudi Arabia should be expanded with references.**

**Response:** Thank you for the comment. We have updated the Introduction section.

**- The final paragraph should clearly define the research gap and justify why studying Saudi families is important.**

**Response:** Thank you for your valuable comment. We revised the justification for EB studies in Arab patients from Saudi Arabia.

**Methodology**

**-Recruitment Criteria: The inclusion and exclusion criteria should be more explicit, especially concerning how patients were diagnosed and classified.**

**Response:** Thank you for your constructive feedback. We have revised the Methodology section to describe the inclusion and exclusion criteria explicitly.

**-Computational Analysis: Provide references for the specific thresholds used in pathogenicity predictions (e.g., CADD score cutoff).**

**Response:** Thank you for the comment. We have now included the details of the computational thresholds used for pathogenicity predictions, including the CADD score cutoff, in the revised Methods section, along with appropriate references.

**Results
- Clearly separate findings related to novel versus known variants to emphasize new contributions.**

**Response:** Thank you for your valuable comment. We have clearly indicated in the Results section which variants are novel.
**- Consider reorganizing the section to first present clinical findings, then genetic results, and finally-computational-predictions.**

**Response:** Thank you for your valuable comment. The Results section now follows the suggested order: clinical findings, followed by genetic analysis, and then computational predictions. **Discussion
- Comparisons with global EB cases are insightful, but more emphasis should be placed on how Saudi genetic variants differ from other populations.
Implications for genetic counseling in consanguineous populations should be elaborated on.**

**Response:** Thank you for your valuable comment. The recommendations were implemented in the revised manuscript. Discussion includes comparisons with previously reported global variants and highlights novel findings in Saudi patients. **Conclusion
- The conclusion should be more concise and explicitly highlight future research directions.**

**Response:** Thank you for your valuable comment. The conclusion summarizes key findings and clearly shows the clinical relevance. **- Mention potential applications of findings, such as early genetic screening programs.**
**Response:** Thank you for your valuable comment. Early screening is briefly mentioned in the Conclusion and Abstract

**Figures & Tables
- Figure 1 (Pedigrees): Improve clarity by ensuring consistent labeling across all families.**

**Response:** Thank you for your valuable comment. Figures of Pedigrees have been revised to enhance clarity and readability, ensuring that the structure and genotype information are presented more clearly to the readers. Please note that with the addition of the figure illustrating the WES workflow, the order of the figures has been adjusted accordingly.
**- Figure 2 (Clinical Presentation): Enhance image descriptions to clarify key features of EB.**

**Response**: Thank you for the suggestion. We have revised the legend and image annotations in Figure 2 to provide more precise descriptions of the key clinical features of epidermolysis bullosa (EB), enhancing the figure’s interpretability.

- **Tables 3 & 4: Consider moving some detailed variant data to supplementary material to improve readability.**

**Response:** Thank you for your comments. We have moved Table 3 to the supplementary information section and incorporated all genotype data directly into the corresponding pedigree figure for clarity and completeness.

**Reviewer #3: The authors have done a good work on the EB families from the middle east. But I have some suggestions and changes for the integrity of the manuscript, I trust in the authors’ capabilities to address all the comments.
\# This study includes families from three ethnic origins i.e., Saudi Arabia, Syria and Yemen, so how the authors focus in the title only on the Saudi Arabian families?
Response:** Thank you for your valuable comment. We corrected the title with appropriate mentions like ‘Middle Eastern families’

**# “This study intends to shed some light on recurrent as well as novel genetic abnormalities that cause EB in the Western region of Saudi Arabia” is a poor presentation please rephrase this sentence e.g., “This study intends to highlight the recurrent as well as novel genetic abnormalities that cause EB in the Western region of Saudi Arabia”.**

**Response:** Thank you for your valuable comment. The corresponding statement has been revised to ‘This study intends to highlight the recurrent and novel genetic abnormalities that cause EB in the Western region of Saudi Arabia’.

**# “Twelve EB families in Saudi Arabia were recruited from clinical settings” this sentence is ambiguous to understand please try to make its sense clearer. What do you mean by clinical settings here?**

**Response:** Thank you for your valuable comment. We corrected the statement, ' Twelve families affected by EB were recruited through dermatology clinics and hospitals in Saudi Arabia, where they received their clinical diagnosis and care.
**# In line 43 (abstract), please correct “classify disease subtypes precisely” as “classified the disease subtypes precisely”.**

**Response:** Thank you for your valuable comment. The phrase has been revised as requested.

**# In line 44 “to identify EB-causing variants” as “to identify EB-causing gene variants”.**

**Response:** Thank you for your valuable comment. The phrase has been revised as requested.

**# In line 47, the authors state that “We identified 11 pathogenic variants”. Were all the variants pathogenic, confirmed by the pathogenicity prediction tools? Otherwise rephrase this sentence and use correct terms regarding classification of the identified variants.**

**Response:** Thank you for your valuable comment. We changed the pathogenic variants to genetic variants.

**#** **Use correct nomenclature regarding the identified frameshift, nonsense, and missense variants nonsense (c.1633C>T, c.1837C>T, c.2005C>T, and c.5888G>A), missense (c.4448G>A, and c.8245G>A). Please follow the variant nomenclature guidelines, how to write a variant that has not been verified through functional studies in the model organisms.**

**Response:** Thank you for the comment. Variant descriptions have been updated to follow HGVS guidelines, including both cDNA and protein-level notation.

**#** **The sentences “The COL7A1 variants included frameshift (c.5924_5927del and c.6268_6269del,), nonsense (c.1633C>T, c.1837C>T, c.2005C>T, and c.5888G>A), missense (c.4448G>A, and c.8245G>A), and splice site (c.6751-1G>A). Additionally, splice site variants were detected in the COL17A1 () and LAMB3 genes (c.8305-1G>A and c.1977-1G>A, respectively)” contains many mistakes please correct.**

**Response:** Thank you for your observation. The sentence has been revised to correct the errors in punctuation, variant formatting, and missing information. The corrected version now follows proper scientific and HGVS nomenclature guidelines.

**# In line 53, “predicted These variants” correct it as “predicted these variants”.**
**Response:** Thank you for your valuable comment. The phrase has been revised as requested.

**# In line 53, the variants are either pathogenic or likely pathogenic but not highly pathogenic, so please correct.**

**Response:** Thank you for your valuable comment. The sentence has been revised as requested.

**# The pedigrees are unreadable and illegible. In some of the pedigrees the lines (horizontal/vertical) are missing, in such situations it is very difficult to reach a conclusion about. To make it more clear and easier for the readers it is advised to write the gene name and variant in the left upper side of the corresponding pedigree. It will be also very helpful to write genotype below each individual in the pedigree. Please follow this article DOI: 10.1007/s10528-025-11087-2 as a guide.**

**Response:** Thank you for your valuable comment. Figure 1 has been revised to enhance clarity and readability, ensuring that the structure and genotype information are presented more clearly to the readers.

**# Add OMIM IDs with the gene and disease names.**

**Response:** Thank you for the suggestion. OMIM IDs have been added alongside all relevant gene and disease names at their first mention in the manuscript, in accordance with standard scientific reporting practices.

**# Write p.Glu1975fs and p.Pro2090fs frameshift variants in their full form. Such as, p.(Glu1975GlyfsTer29) and p.(Pro2090TrpfsTer8). Also please use uniform nomenclature for c.5924_5927delAACG, c.5924_5927del and other variants like c.1633C>T (p. Gln545Ter), c.5888G>A (p. Trp1963X), c.2005C>T (p.Arg669*), c.1837C>T (p.Arg613*) etc., throughout the manuscript.**

**Response:** Thank you for your valuable comment. The frameshift variants have been updated to their full forms and consistent HGVS-compliant nomenclature has been applied to all variants throughout the manuscript.

**# In line 140, it will be better to use GRCh37/hg19 in place of only hg19, because it has already been used in line 211.**

**Response:** Thank you for the observation. “hg19” in line 140 has been updated to “GRCh37/hg19” for consistency with the terminology used in line 211.

**# In line 147, add the reference for ACMG guidelines (DOI: 10.1038/gim.2015.30).**

**Response:** Thank you for the valuable comment. The reference for the ACMG guidelines (Richards et al., 2015) has been added to the text at line 147 as requested.

**#** **The COL17A1 nonsense variant c.1394G>A; p.(Trp465X) is not mentioned in the text on page 6 under the heading “Genetic Analysis”.**

**Response:** Thank you for pointing this out. The COL17A1 nonsense variant c.1394G>A; (p.Trp465*) has now been added to the “Genetic Analysis” section on page 6 to reflect the findings identified in Family 6 accurately.

**# Write the gene names in italics throughout the manuscript, e.g., in lines 247 etc.**

**Response:** Thank you for your valuable comment. All gene names have been italicized throughout the manuscript, including in line 247 and other relevant sections.

**# The “in text-citations” format is not correct, e.g., line 70-- (1) (2) (3), line 93-- (12) (13), line 97-- (14) (12). Write in a correct format such as (1-3), (12, 13), (12, 14).**
**Response:** Thank you for your observation. All in-text citations have been corrected and reformatted to comply with the appropriate citation style.

**# In lines 150, 151… segregation of the variant with disease in other affected family members. It will be more appropriate to state “segregation of the variant with disease in other family members”, because the variant may segregate randomly in any of the siblings in heterozygous/homozygous form.**

**Response:** Thank you for your valuable comment. The sentence has been revised as requested.

**# Use proper reference/URL with the softwares or tools used in the text, e.g., SnapGene version 6.0.2, CADD, FATHMM and SpliceAI etc.**

**Response:** Thank for your comment. We add the reference for the software and tools as following:

1. SnapGene Version 6.0.2

Reference: SnapGene Version 6.0.2 Release Notes. SnapGene. January 12, 2022.

URL: https://www.snapgene.com/updates/snapgene-6-0-2-release-notes

2. CADD (Combined Annotation Dependent Depletion)

Reference: Kircher M, Witten DM, Jain P, et al. A general framework for estimating the relative pathogenicity of human genetic variants. Nature Genetics. 2014;46(3):310–315. doi:10.1038/ng.2892.

URL: https://cadd.gs.washington.edu/

3. FATHMM (Functional Analysis Through Hidden Markov Models)

Reference: Shihab HA, Gough J, Cooper DN, et al. Predicting the functional, molecular, and phenotypic consequences of amino acid substitutions using hidden Markov models. Human Mutation. 2013;34(1):57–65. doi:10.1002/humu.22225.

URL: https://www.researchgate.net/publication/336909741

4. SpliceAI

Reference: Jaganathan K, Kyriazopoulou Panagiotopoulou S, McRae JF, et al. Predicting splicing from primary sequence with deep learning. Cell. 2019;176(3):535–548.e24. doi:10.1016/j.cell.2018.12.015.

URL: https://spliceailookup.broadinstitute.org/

**#** **In line 179, replace “variant forms” with “mutant forms”.**

**Response:** Thank you for your valuable comment. The phrase “variant forms” in line 179 has been replaced with “mutant forms” as recommended.

**# In line 187, the authors have simply mentioned that “sixteen patients from 12 different families were clinically diagnosed in the clinic” please mention the name of the clinic/s in order to increase credibility and trustworthiness of the research work.**

**Response:** Thank you for your valuable comment. We updated the methodology section by mentioning the dermatology clinic.

**# In line 189, please write the families numbers (11 8 and 12) in a sequence such as 8, 11, and 12. Here it is difficult to confirm that which family belongs to Syria and which one is of Yemeni origin?**

**Response:** Thank you for your valuable comment. The sentence has been revised to clearly indicate that Families 8 and 12 are of Yemeni origin, while Family 11 is of Syrian origin.

**# In line 191, the authors have mentioned that “Pedigree analysis (Fig 1) suggested an
autosomal recessive mode of inheritance” but family 12 has an autosomal dominant inheritance pattern(haven't mentioned).**
**Response:** Thank you for your comment. The text has been revised and updated to reflect the inheritance patterns observed in the study accurately. Specifically, we have clarified that Family 12 exhibits an autosomal dominant inheritance pattern, while most other families follow an autosomal recessive mode.

**# In line 194, replace (Fig 2) with Figure 2.**

**Response:** Thank you for your comment. However, we are following the PLOS ONE journal style, which specifies the use of the abbreviation “Fig” rather than writing out “Figure.”

**# In table 1, correct the font style in column 7.**

**Response**: Thank you for the observation. The font style in column 7 of Table 1 has been corrected as requested.

**# In table 2, correct the gene symbol COL17A as COL17A1.**
**# For each variant classification based on the ACMG/AMP criteria, write the evidences such as very strong (PVS1), Strong (PS1-4), moderate (PM1-6) or supporting (PP1-5) to be pathogenic or likely pathogenic.**

**Response:** Thank you for the comment. The identified splice-site variants in **LAMB3 (c.1977-1G>A) and COL7A1 (c.8305-1G>A and c.6751-1G>A)** are classified as **pathogenic** based on ACMG/AMP guidelines. All fulfill **PVS1** (canonical splice site, LOF mechanism), **PM2** (absent in population databases), and **PP3** (predicted impact by SpliceAI). These combined criteria support their pathogenic classification, and this has been clarified in the revised manuscript.

**# Correct the heading of table 4, these are the “details of mutations identified in EB patients” add at the end “so far”.**

**Response:** Thank you for your valuable comment. The heading of Table 4 has been revised as recommended.

**# Correct the sentence “The current study sheds light on the genetic makeup of EB in Saudi patients by detecting causative variants in the COL7A1, COL17A1, and LAMB3 genes” here not the genetic makeup of EB but the genetic makeup of Saudi EB patients. So, correct the above sentence. Also replace “sheds light on” with “highlights”.**

**Response:** Thank you for the valuable comment. The sentence has been revised to:

“The current study highlights the genetic makeup of Saudi EB patients by detecting causative variants in the COL7A1, COL17A1, and LAMB3 genes,” as suggested.

**# It will be more suitable to use “variant” in place of “mutation”.**

**Response:** Thank you for your valuable comment. The term “mutation” has been replaced with “variant” throughout the manuscript to align with current scientific terminology.

**\# No description about the families, consanguineous or nonconsanguineous and how many individuals are present each family etc, which is mandatory to give the detailed description of each family to the readers.**

**Response:** Thank you for your valuable comment. We have added details regarding consanguinity in the text and have updated Figure 1 to clearly represent all participants enrolled in the study, including the number of individuals per family and their relationship structure.

**# In lines 360-364 “A total of ten mutations were identified in COL7A1, including three missense mutations (c.4448G>A and c.8245G>A), three frameshift mutations (c.5924_5927delAACG, c.6268_6269del, c.1837C>T), two splice site mutations (c.8305-1G>A, c.6751-1G>A), and two truncating mutations (c.1633C>T, c.2005C>T and c.5888G>A)”. This statement is incorrect because:
Missense: 2 --------(c.4448G>A and c.8245G>A)
Frameshift: 2-------(c.5924_5927del, c.6268_6269del)
Nonsense/truncating: 4------- c.1633C>T; p.(Gln545Ter), c.1837C>T; p.(Arg613Ter), c.2005C>T; p.(Arg669Ter), c.5888G>A; p.(Trp1963Ter).
Splice site: 2--------(c.6751-1G>A; p.?, c.8305-1G>A ;p.?), so correct the above statement.**

**Response:** Thank you for the correction. The statement has been revised to reflect the number and type of variants as outlined accurately.

**# The discussion is about the comparison of your results as in this case “the patients’ clinical phenotypes, number of patients in the families, their gender, geography and the identified variants in each family” are logically compared with the previous literature. But here in this manuscript this critical point seems to be missing. The authors are advised to compare their results with those present in table 4.**

**Response:** Thank you for your valuable comment. We compared the global data mutations with our patient mutation data in the discussion section.
